# Coaxial Electrospray of Ranibizumab-Loaded Microparticles for Sustained Release of Anti-VEGF Therapies

**DOI:** 10.1371/journal.pone.0135608

**Published:** 2015-08-14

**Authors:** Leilei Zhang, Ting Si, Andrew J. Fischer, Alan Letson, Shuai Yuan, Cynthia J. Roberts, Ronald X. Xu

**Affiliations:** 1 Department of Biomedical Engineering, The Ohio State University, Columbus, Ohio, 43210, United States of America; 2 School of Engineering Science, University of Science and Technology of China, Hefei, Anhui, 230027, People’s Republic of China; 3 Department of Neuroscience, The Ohio State University, Columbus, Ohio, 43210, United States of America; 4 Department of Ophthalmology, The Ohio State University, Columbus, Ohio, 43210, United States of America; The University of Tennessee Health Science Center, UNITED STATES

## Abstract

Age-related macular degeneration (AMD) is the leading cause of vision loss and blindness in people over age 65 in industrialized nations. Intravitreous injection of anti-VEGF (vascular endothelial growth factor) therapies, such as ranibizumab (trade name: Lucentis), provides an effective treatment option for neovascular AMD. We have developed an improved coaxial electrospray (CES) process to encapsulate ranibizumab in poly(lactic-co-glycolic) acid (PLGA) microparticles (MPs) for intravitreous injection and sustained drug release. This microencapsulation process is advantageous for maintaining the stability of the coaxial cone-jet configurations and producing drug-loaded MPs with as high as 70% encapsulation rate and minimal loss of bioactivitiy. The utility of this emerging process in intravitreous drug delivery has been demonstrated in both benchtop and *in vivo* experiments. The benchtop test simulates ocular drug release using PLGA MPs encapsulating a model drug. The *in vivo* experiment evaluates the inflammation and retinal cell death after intravitreal injection of the MPs in a chick model. The experimental results show that the drug-load MPs are able to facilitate sustained drug release for longer than one month. No significant long term microglia reaction or cell death is observed after intravitreal injection of 200 μg MPs. The present study demonstrates the technical feasibility of using the improved CES process to encapsulate water-soluble drugs at a high concentration for sustained release of anti-VEGF therapy.

## Introduction

In recent years, more and more protein drugs, such as recombinant human proteins and monoclonal antibodies, are developed with the advancement of biotechnology [1,2]. However, many protein drugs have a relatively short half-life and therefore require repetitive administration at a high frequency [2]. One example is intravitreous injection of anti-VEGF (vascular endothelial growth factor) therapies for the treatment of age-related macular degeneration (AMD). AMD is the leading cause of vision loss and blindness in people over age 65 in industrialized nations [3–5]. It can be divided into two categories: nonexudative AMD and exudative AMD. The exudative AMD is characterized by choroidal neovascularization (CNV) and retina pigment epithelium (RPE) detachments [6]. Although the exudative AMD accounts for only 10% to 20% of AMD cases, it causes 80% to 90% of cases with severe vision loss related to AMD [7]. VEGF plays a very important role in the development of AMD, especially exudative AMD. Intravitreous injection of anti-VEGF therapies, such as ranibizumab (trade name: Lucentis), is a widely accepted treatment for neovascular AMD [8]. However, this procedure recommends monthly injection because of the short half-life (usually 2–5 days) [5–6]. The repetitive intravitreous injection increases the risk of multiple complications and adverse reactions, such as endophthalmitis, retinal detachment, and iatrogenic traumatic cataract [9–11].

To lengthen the drug release time and reduce the frequency of repetitive administration, protein drugs are encapsulated in biodegradable microparticles (MPs) [7–8] or nanoparticles (NPs) [9–10]. Commonly used carrier materials for these MPs and NPs include liposome, albumin, polylactide (PLA), and poly-lactic-co-glycolic acid (PLGA). PLGA is an FDA approved biodegradable and biocompatible material for implantation applications. The release time of PLGA MPs can be programmed by controlling the particle morphology, the molecular weight of PLGA polymer, and the particle composition. Emulsification is one of the most commonly used microencapsulation methods for protein drugs. Although the process is simple, it has multiple disadvantages, such as a low encapsulation rate for water-soluble cargos, a broad size distribution, and possible denaturation and aggregation of the encapsulated bioactive cargos [12].

To overcome the above-mentioned limitations, we propose to use an improved coaxial electrospray (CES) process to encapsulate ranibizumab in PLGA MPs for intravitreous injection and sustained drug release. CES, also known as coaxial electrohydrodynamic atomization, is an emerging microencapsulation technique [13,14]. It can be potentially used to encapsulate protein drugs with high encapsulation rate, uniform size distribution, and with protection of protein bioactivities. In this study, ranibizumab encapsulated MPs are fabricated by a CES process. The encapsulation rate and the release profile of the produced MPs are tested by *in vitro* experiments. The inflammatory response and cell death after intravitreous injection of the MPs is examined in an *in vivo* chick model. The chick model is used for the *in vivo* study for several reasons. First, a chick has much larger eyes than a rodent model, more convenient for experimental exploration and manipulation [15]. Second, a chick has a much smaller intraocular lens than a rodent model, which is much easier for intravitreal injection. Third, a chick model is less expensive than a rodent model, especially for the newly hatched chicks. Moreover, a chick model has already been used for studying retinal damage [16–18]. Since microglia responses fast to acute retinal damage, its proliferation is examined after injection of MPs [19,20]. After the microglia study, the TUNEL assay is used to further investigate MP-induced cell death [21,22]. Apoptosis and necrosis are two major mechanisms contributing to the retina cell death [23,24]. Necrosis happens when the cell fails to maintain homeostasis, which is usually related to physical damage. Apoptosis, also called programmed cell death, is often characterized by chromatin condensation or nuclear shrinkage. Considering that intravitreous drug delivery is associated with the risk of infection, inflammatory response, transretina migration of particles, and damage to ocular structure [25], it is appropriate to identify individual cell apoptosis by the TUNEL assay after injection of MPs [26,27].

## Materials and Methods

### Materials

Poly (lactic-co-glycolic acid) (PLGA) (50:50, *M*_*w*_ = 12,000) was purchased from Boehringer Ingelheim (Ingelheim, Germany). Ranibizumab was a generous gift from Genentech (San Francisco, CA). Dichloromethane (DCM), acetonitrile, ethylene glycol (EG), and Dimethyl sulfoxide (DMSO) were purchased from Fisher Scientific (Newton, NJ). The fluorescence dye 1,1'-Dioctadecyl-3,3,3',3'-tetramethylindocarbocyanine perchlorate (‘DiI’, D-282) was purchased from Invitrogen (Grand Island, NY) and Nile red from Sigma-Aldrich (St. Louis, MO). Rhodamine-6G was purchased from Acros organics (Fisher Scientific Newton, NJ). The Enzyme-linked immunosorbent assay (Elisa) plate was purchased from Greiner Bio-one (Monroe, NC).

### Experimental CES device

**Fig 1:**
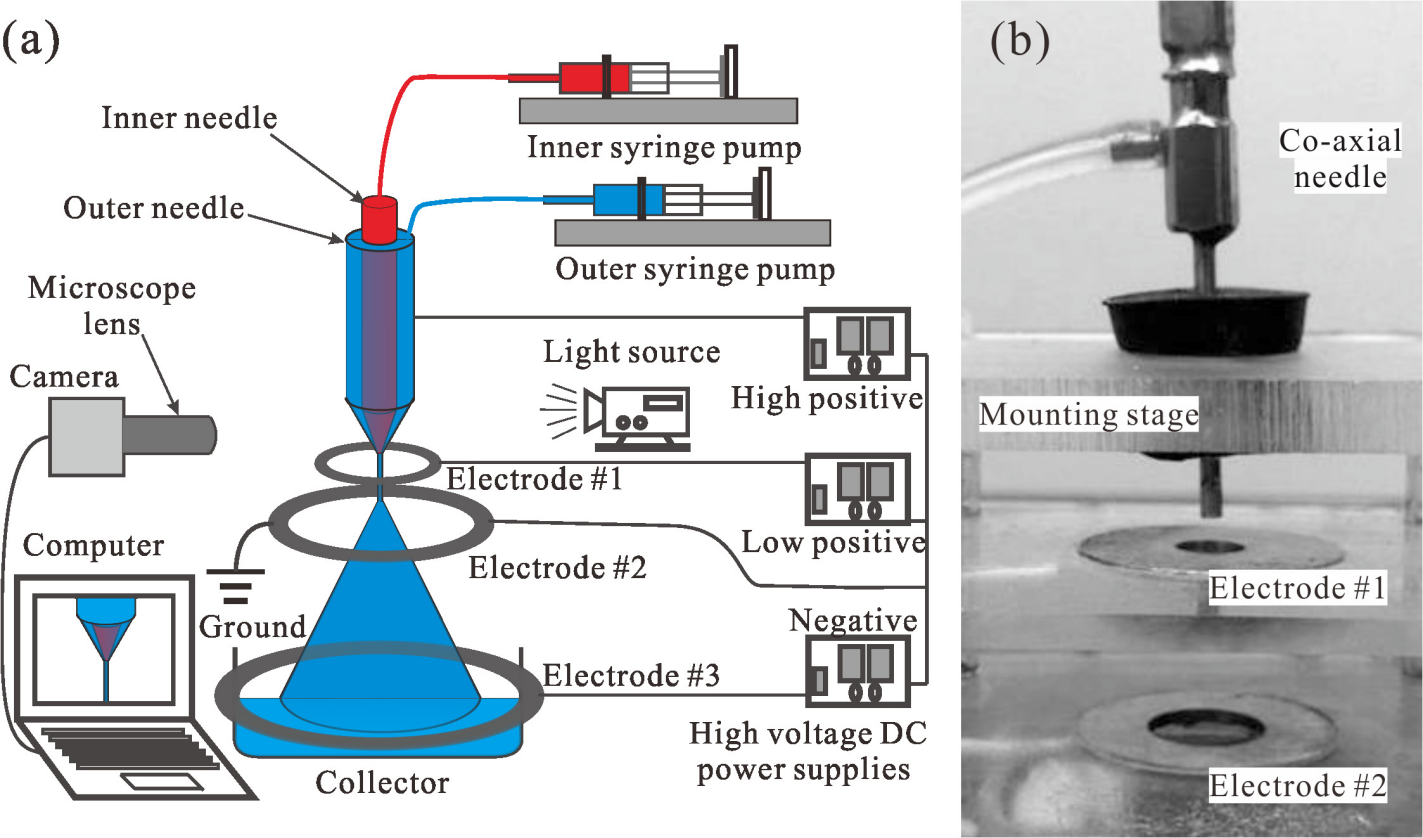
Experimental CES system. (a) schematic of the experimental setup for the CES process, and (b) corresponding picture of the coaxial needle and the first two ring electrodes.

A schematic of the experimental setup for the CES method is shown in Fig 1. The key component is a stainless steel coaxial needle assembly that consists of an outer needle of gauge 14 and an inner needle of gauge 24 (Fig 1B). Two liquids are injected into the outer and the inner needles, with the flow rates controlled by two syringe pumps (KD scientific Inc., MA), respectively. The coaxial needle and the electrodes are connected to high voltage DC power supplies (Gamma high voltage research, Inc., FL).

In a common CES device, a ring electrode (negative or ground) is placed at a specific distance below the coaxial needle assembly (positive). Under the elevated electric field, a Taylor cone is formed at the tip of the coaxial needle, and the coaxial jet of the inner and the outer liquids is eventually broken into multi-layer droplets due to flow instability. Practically, it is difficult to stabilize the Taylor cone and the coaxial jet, owing to the large separation between the two electrodes and the evaporation of solvent during the process. To solve the stability problem, an improved CES device with three ring electrodes is designed, as shown in Fig 1. This design includes a positively charged coaxial needle at higher voltage and a positively charged ring electrode #1 at a lower voltage. The ring electrode #1 is placed 1~3 mm below the coaxial needle to stabilize the Taylor cone. The addition of the ring electrode #1 introduces another problem that the droplets partially fly back and stick to the electrode. Therefore, a second ring electrode (electrode #2) is placed between the ring electrode #1 and the bottom ring electrode #3. Electrode #2 is grounded and the electrode #3 is negatively charged so that the droplets are accelerated towards the collecting container at the bottom of the CES device. The relative positions of the coaxial needle and the electrodes can be adjusted by a mounting stage. In most experiments, the vertical distances from the coaxial needle tip to the electrode #1, #2, #3 are chosen to be 2.5 mm, 33 mm, 134 mm, sequentially. More importantly, this design of multi-level electrodes can efficiently reduce the applied voltage of each electrode, which would protect the bioactivity of encapsulated materials like drugs and proteins. To monitor the fabrication process, a 3 kHz strobe flashlight is placed on one side of the coaxial needle and the electrodes, and a microscopic lens combined with a charge-coupled device camera (Allied vision technologies, Inc., MA) is placed on the other side. The dynamic images of cone-jet configurations in the CES process can be easily observed in a computer for process optimization.

### MP preparation and characterization

To fabricate MPs by the improved CES process, the outer and the inner liquids are prepared in advance. To prepare the outer liquid, PLGA is dissolved into 50/50 v/v DCM and acetonitrile mixture with a concentration of 60 mg/mL. Fluorescence dye (Nile red or DiI) is added in the PLGA solution for further characterization of MPs. To prepare the inner liquid, ranibizumab is dissolved into 50/50 v/v saline and EG mixture with final concentration of 1.2 mg/mL. The release experiment uses Rhodamine-6G as the model drug. To prepare Rhodamine-6G encapsulated MPs, Rhodamine-6G is dissolved into 50/50 v/v saline and EG mixture with a final concentration of 10 mg/mL as the inner liquid. For fundamental verification, the mixture of 50:50 v/v distilled water and EG is used as the inner liquid.

The morphology of the produced PLGA MPs is characterized by a Hitachi S-3000 scanning electron microscope (SEM) (Hitachi High Technologies America, Inc., Pleasanton, CA) after centrifugation. The size distribution of MPs is characterized by a Dynamic laser scattering device (‘DLS’, BI-200SM, Brookhaven Instruments Corporation, NY). The core-shell structure of PLGA MPs is verified under a Leica TCS SL confocal microscopy (Leica Microsystems Inc., Buffalo Grove, IL).

Elisa tests are carried out to evaluate ranibizumab’s bioactivity after exposure to high voltage during electrospray, mechanical force, and freeze drying, respectively. To test ranibizumab’s bioactivity after electrospray, 10 μL ranibizumab at 10 mg/mL is dissolved in 10 mL saline and sprayed at a flow rate of 2 mL/h for 15 minutes in an electric field from 0 kV to 9 kV at an incremental step of 1 kV. To test the effects of mechanical force and freeze drying on ranibizumab’s bioactivity, 15 μL ranibizumab at 10 mg/mL is dissolved in 5 mL saline. Half of the liquid is freeze-dried by LYPH LOCK for 36 hours. The other half is emulsified by an Omini mixer homogenizer at 20,000 rpm for 3 minutes. The bioactivities of all the samples are tested by an Elisa assay right after the experiment.

### Drug loading and *in vitro* release

The encapsulation rate of ranibizumab-loaded MPs is characterized by an Agilent UV-VIS spectrophotometer (Agilent Technologies, CA). The standard curve is obtained in advance using a baseline solution that dissolves 80 mg PLGA in 2 mL DMSO. As ranibizumab is added to the baseline solution to reach multiple concentration levels from 50 μg/mL to 200 μg/mL, the absorbance at each concentration level is measured at the wavelength of 290 nm in order to calculate the standard curve. To evaluate the encapsulation rate of the ranibizumab-loaded MPs, 80 mg of freeze dried MPs is dissolved in 2 mL DMSO and the absorbance is tested by a UV-VIS spectrophotometer. The encapsulation rate is calculated by finding the concentration level on the standard curve that corresponds to the measured absorbance.

Rhodamine-6G is used as a model drug to investigate the *in vitro* release profile of drug-loaded PLGA MPs because Rhodamine-6G has been widely used for similar applications in ocular drug delivery [28–30]. For the control group, 250 μg of Rhodamine-6G is dissolved in 25 mL saline to form a final concentration of 10 μg/mL. For the sample group, 30 mg PLGA MPs is placed in a dialysis membrane (model No. 88242, Fisher Scientific). The membrane is submerged in 25 mL saline solution. Both the control and the sample are placed in separate beakers covered by aluminum foil and incubated in a 37°C mineral oil bath. At 1, 2, 3, 4, 6, 8, 10, 12, 15, 17, 20, 23, and 26 days after incubation, 300 μL of the control and the sample solutions are collected and stored in centrifuge tubes at -80°C for future analysis. All the measurements are acquired in triplicate.

### *In vivo* inflammation test

#### Animal model preparation

Newly hatched leghorn chicks are obtained from the Department of Animal Sciences at The Ohio State University. Postnatal chicks are kept on a cycle of 12 h light and 12 h dark (lights on at 8:00 AM). Chicks are housed in a stainless steel brooder at about 25°C with the supply of water and chick starter (Purina, St. Louis, MO) ad libitum [31]. The use of the animals follows an established protocol approved by The Ohio State University Institutional Animal Care and Use Committee (protocol No: 2009A0139).

#### Intravitreal injection

DiI encapsulated MPs are used for the intravitreous injection. The size of the MPs ranges from 1 μm to 2 μm. Before injection, the MPs are freeze dried and stored in a glass bottle wrapped with aluminum foil at -20°C. Chicks are anesthetized by inhalation of 2.5% isoflurane in O_2_ at a flow rate of 1.5 L/min for 10 seconds. The gas is kept on during the whole injection which is usually 2–3 minutes per chick. Injections are made with a 25 μL Hamilton syringe and a 26G needle with a cutting tip. The needle is consistently inserted into the dorsal quadrant of the vitreous chamber through the pars plana and dorsal eye lid, which is sterilized with iodine solution after each injection. During all the experiments, the left eyes of chicks are considered as treated eye by injecting “test” compound and the right eyes are injected with saline plus BSA as a control. The separated syringes are used for the treated eyes and controlled eyes in the whole experiments [32,33].

Fifteen chicks are involved in the intravitreal injection. Three chicks are injected with 20 μL of 10 mg/mL ranibizumab in each of the treatment eyes. The same volume of saline with 0.05 mg/mL BSA is injected into each of the control eyes. Another twelve chicks are divided into three even groups and injected with 20 μL of 0.1 mg/mL, 1 mg/mL, and 10 mg/mL MPs respectively. The same volume of saline with 0.05 mg/mL BSA is injected into each of the control eyes. The three chicks with intravitreous injection of ranibizumab are sacrificed one day after injection. Half of the chicks after intravitreous injection of MPs are sacrificed one day after the treatment. The other half are sacrificed twelve days after injection.

#### Tissue dissection, fixation and sectioning

Chicks are euthanized by carbon dioxide inhalation for 5 minutes. Eyes are removed from the orbit. Most of the connective tissues and muscles are removed. The enucleated eyes are hemisected equatorially and the vitreous gel of treated eyes is collected from the posterior eye cup. After that, each of the vitreous samples is placed on a microscopic slide double ringed by rubber cement, sealed with a cover slide, and stored at -20°C for further use. The isolated posteriors of the eye cups are used for cryosectioning preparation. Samples are fixed for 30 minutes at room temperature in 4% paraformaldehyde plus 3% sucrose in 0.1 M phosphate buffer, pH 7.4. The samples are then washed three times in PBS (0.05 M phosphate buffer, 195 mM NaCl, pH 7.4), cryoprotected in PBS plus 30% sucrose overnight, soaked in embedding medium (O. C. T. compound; Tissue-Tek; Elkhart, IN) for 10 minutes at -80°C, and freeze-mounted onto aluminum sectioning blocks. Vertical sections of 12 μm thick are consistently cut in the nasotemporal plane, and thaw-mounted onto SuperFrost Plus slides (Fisher scientific). Sections of the control and the treatment eyes from the same chick are placed consecutively on each slide to ensure equal exposures to reagents and facilitate comparison of control and treated retinas. Sections are stored at -20°C for use [33–35].

#### Immunocytochemistry analysis

Standard immunocytochemical techniques are used as described previously [32,33,36]. Briefly, sections with the control and the treated retinas placed in pairs are ringed with rubber cement to form a well for antibody solutions. After the rubber cement is dried, the sections are washed three times in PBS, covered with primary antibody solution, and incubated overnight at room temperature in a humidified chamber. On the second day, the slides are washed three times in PBS, covered with secondary antibody solution, and incubated for at least 1 hour at room temperature in a humidified, dark chamber. The procedure is repeated in the case of second-labeling or third-labeling. Finally, the slides are washed three times in PBS, the rubber cement is removed, and the slides are cover slip mounted in a solution of 4:1 v/v glycerol and water.

In ranibizumab injection, triple-labeling is used to stain ranibizumab, CD45, and Draq5. Goat anti-human IgG (Fab) at 1:1000 (Fisher scientific) and donkey anti-goat-Alexa488 (Invitrogen) at 1:1000 in PBS plus 0.2% Triton X-100 and 0.01% NaN_3_ are used as primary and secondary antibody for ranibizumab labeling. For CD45 labeling, mouse anti-CD45 at 1:100 (Cedi Diagnostic) and goat anti-mouse-Alexa568 (Invitrogen) diluted to 1:1000 are used. Moreover, the slides are also stained with DRAQ5 diluted to 1:2000 (Biostatus Limited) to label nuclear DNA.

In MPs injection, double-labeling is used to stain CD45 and Draq5. For CD45 labeling, mouse anti-CD45 at 1:100 (Cedi Diagnostic) and goat anti-mouse-Alexa488 (Invitrogen) diluted to 1:1000 are used. The slides are also stained with DRAQ5 diluted to 1:2000 (Biostatus Limited) to label nuclear DNA.

TUNEL method is used to detect the fragmented DNA in dying cells. The *In Situ* Cell Death Kit (TMR red; 1215679910) is purchased from Roche Applied Science. The experiment followed the manufacturer’s instructions.

#### Microscopic and photographic imaging

A Leica DM5000B microscope equipped with epifluorescence and a 12 megapixel Leica DC500 digital camera are used to take photomicrographs. Images are optimized for color, brightness, and contrast, and double-labeled or triple-labeled images overlaid by using Adobe Photoshop software. To avoid the possibility of region-specific differences within the retina, all tissues in the same experiment are processed at the same time using same-batch preparation of all reagents and the treated and control retinas from the same chick is placed consecutively (within 3mm) on glass slides [37,38].

## Results and Discussion

Formation of a stable cone-jet mode in CES process is significant for producing core-shell structured MPs. In our previous work, we performed a systematic experimental and theoretical study on optimizing the process control of the CES [13]. The results indicated that as the applied voltage between the coaxial needle and the ring electrodes increased by keeping the other operation parameters constant, the following four operating modes could be observed consecutively: dripping mode, coning mode, stable cone-jet mode and multi-jet mode. With different material properties, the critical applied voltages between these modes might change. In this work, we also observe the four flow modes and identify their critical applied voltages for fixed group of other operation parameters. Particularly, we mainly focus on forming the stable cone-jet mode, which is considered as the mostly suitable mode in fabricating monodisperse MPs with fine core-shell structures [14].

In the improved CES device, the flow mode transition is mainly determined by the applied electric voltages of the coaxial needle and the ring electrodes #1 (high and low positive). Once a stable cone-jet mode is formed, the cone can maintain stable in a wide range of operation parameters, such as the overall applied voltage, flow rate, and the distant between electrodes, because of the design of three parallel ring electrodes. Therefore, in this device the unwanted perturbations during the CES process can be effectively prevented for the mass production of drug-loaded PLGA MBs. Furthermore, for a stable cone-jet mode, the collection of the resultant MPs is mainly attributed to the applied electric voltage of the ring electrode #3 (negative). For a moderate negative voltage, most of MPs can be collected at the bottom of the device for further processing. However, if this voltage is relatively small, the MPs after the breakup of the coaxial jet may adhere to the ground electrode #2, resulting in a waste of drugs; while if this voltage is extremely large, the acceleration of the MPs during the moving period may cause high velocities of the MPs above the collector, which would affect the morphology of the resultant MPs. Therefore, prior to the mass production of drug-loaded PLGA MPs, the group of operation parameters in the CES process is optimized to ensure a maximum loading capacity of drugs.

**Fig 2:**
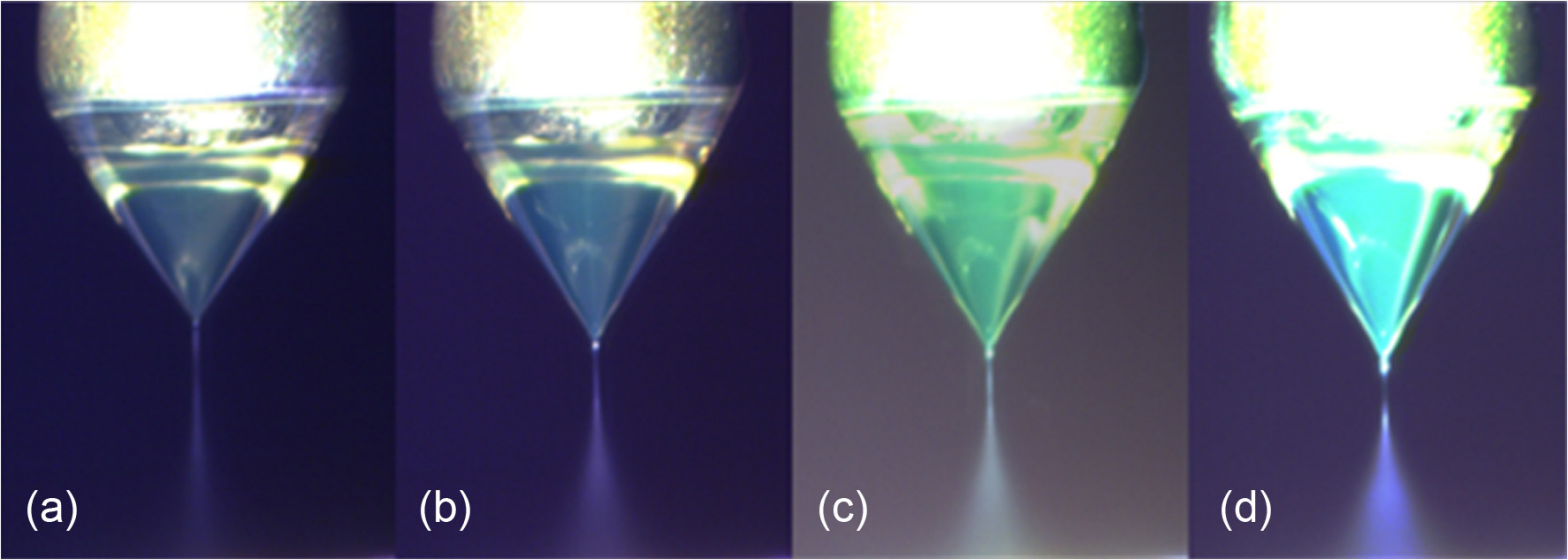
The experimental images of the stable cone-jet mode for increasing outer liquid flow rate of (a) 0.5 mL/h, (b) 0.7 mL/h, (c) 0.9 mL/h, (d) 1.1 mL/h. Inner solution: 50:50 v/v distilled water and EG, 0.5 mL/h; Outer solution: 60 mg/mL PLGA in 50:50 v/v dichloromethane and acetonitrile. The applied electric voltages are +5 kV, +3.5 kV, 0 kV, and -5 kV on the coaxial needle, the ring electrode #1, the ground electrode #2 and the bottom ring electrode #3, respectively.

Several typical images of the stable cone-jet mode in experiments showing the cone-jet morphology as a function of the outer liquid flow rate are presented in Fig 2. The cone maintains stable and the interface between the inner and the outer liquids of the cone can be clearly identified, which implies the success of the microencapsulation. As the outer liquid flow rate increases, the cone becomes elongated and the coaxial jet at the vertex of the cone becomes short and thick. The movement of the resultant microparticles in the atomization process is very fast so that an envelope of the moving MPs is obtained in these images of long exposures. The difference between these envelopes predicts the size variation of resultant MPs in different conditions to an extent. For given liquids and a CES device, the size of resultant MPs after the coaxial liquid jet breakup is mainly determined by the operation parameters including the applied electric voltages, the inner and the outer liquid flow rates. We have found that the mean diameter of the microparticles produced by the breakup of the coaxial liquid jet decreases as the applied electric voltages increase, while it is positively correlated with the inner and the outer liquid flow rates [13].

**Fig 3:**
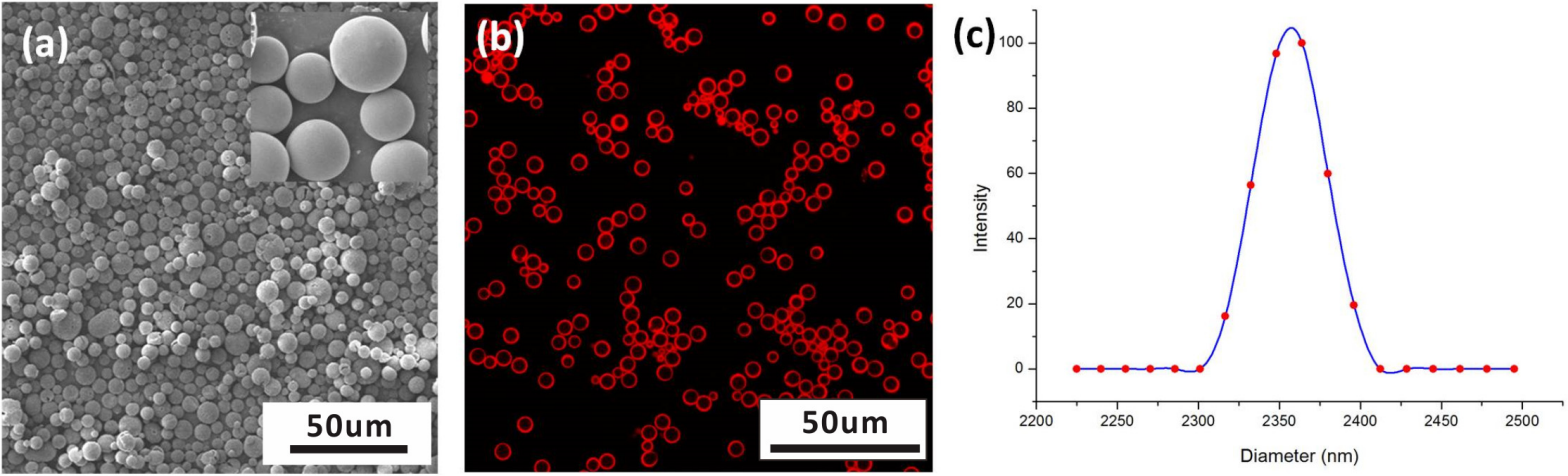
Characterization of core-shell structured PLGA MPs: (a) SEM imaging, (b) Confocal imaging, and (c) size distribution. Inner solution: 50:50 v/v distilled water and EG, 2 mL/h; Outer solution: 60mg/mL PLGA in 50:50 v/v dichloromethane and acetonitrile with small amount of Nile red, 0.5 mL/h. The applied electric voltages are +6 kV, +4 kV, 0 kV, and -6 kV on the coaxial needle, the ring electrode #1, the ring electrode #2 and the bottom ring electrode #3, respectively.

We further produced the core-shell structured PLGA MPs with fine morphology and narrow size distribution in the stable cone-jet mode. Fig 3(A) shows the SEM image of the MPs. The confocal microscopic image in Fig 3(B) shows that the MPs fabricated by CES have a very clear core-shell structure. The thin shell of the PLGA MPs is stained by Nile red. The fabricated MPs have a mean diameter of 2.36 μm with a standard deviation of 0.019 μm (Fig 3C). During this CES process, the outer and inner flow rates are set to be 2 mL/h and 0.5 mL/h, respectively. The applied electric voltages in the experiment are +6 kV, +4 kV, 0 kV, and -6 kV on the coaxial needle, the ring electrode #1, the ring electrode #2 and the bottom ring electrode #3, respectively.

**Fig 4:**
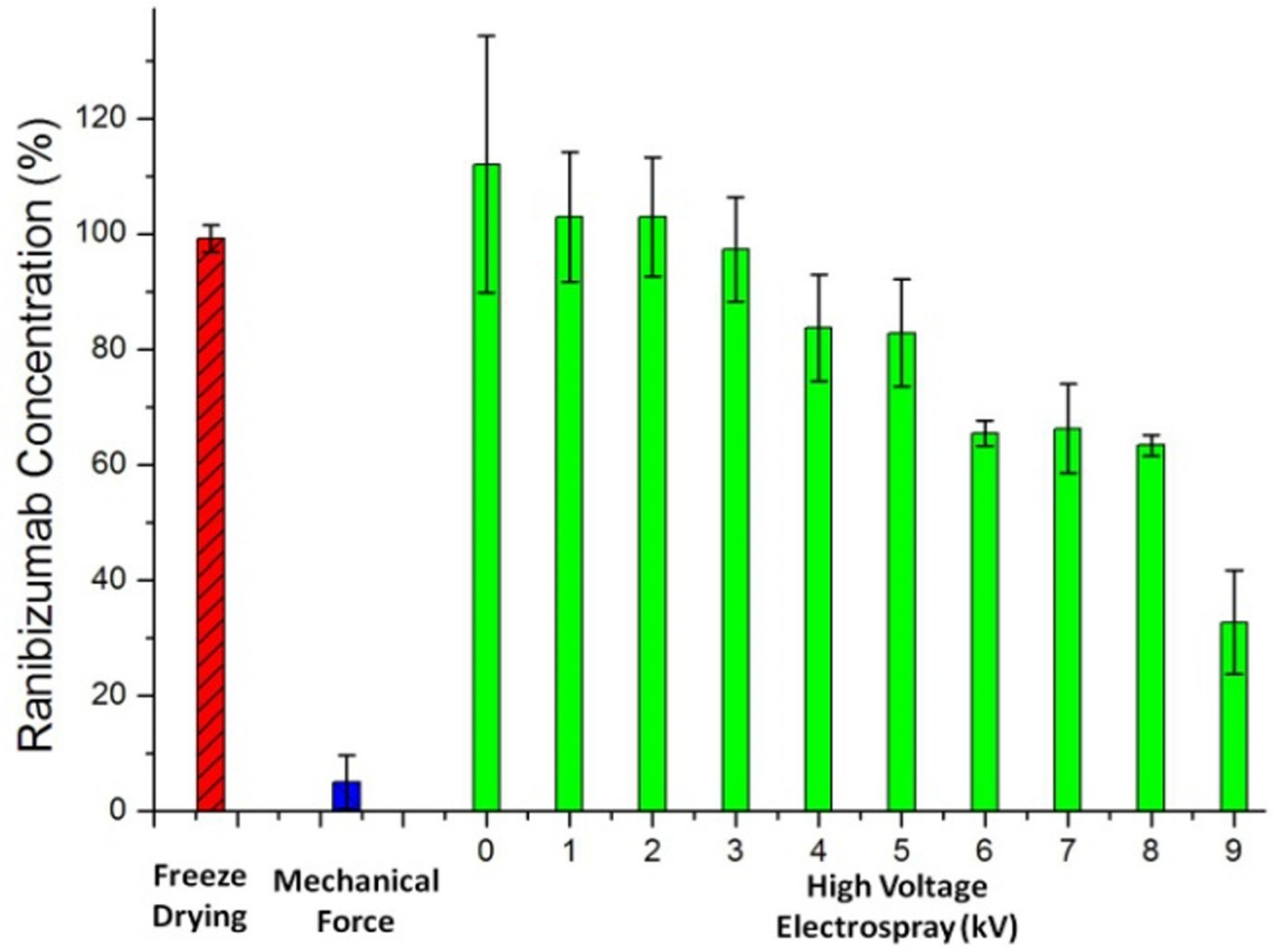
Comparison of ranibizumab concentration after freeze drying (red bar), mechanical force (blue bar), and high voltage electrospray from 0 kV to 9 kV (green bars). The data are presented as mean ± SD from three consecutive ELISA tests.

Fig 4 compares the ranibizumab binding affinity levels acquired by ELISA after freeze drying, mechanical force, and electrospray at different voltage levels from 0 kV to 9 kV. In the experiments, the averaged concentration levels of ranibizumab are obtained from three consecutive ELISA tests and the error bars represent the standard deviations (SD). These results provide relative comparison of ranibizumab bioactivities after various processes. According to the figure, mechanical damage is the primarily reason for the loss of bioactivity for ranibizumab. Since a traditional emulsification process involves vigorous rotating and mixing actions, the mechanical force causes protein denaturation and limits its application in protein drug encapsulation. In comparison, the bioactivity of ranibizumab remains consistent after freeze drying and is above 80% after electrospray at a voltage below 5 kV. This result indicates that CES followed by freeze drying can effectively protect the bioactivity of the protein drugs in a microencapsulation process.

**Fig 5:**
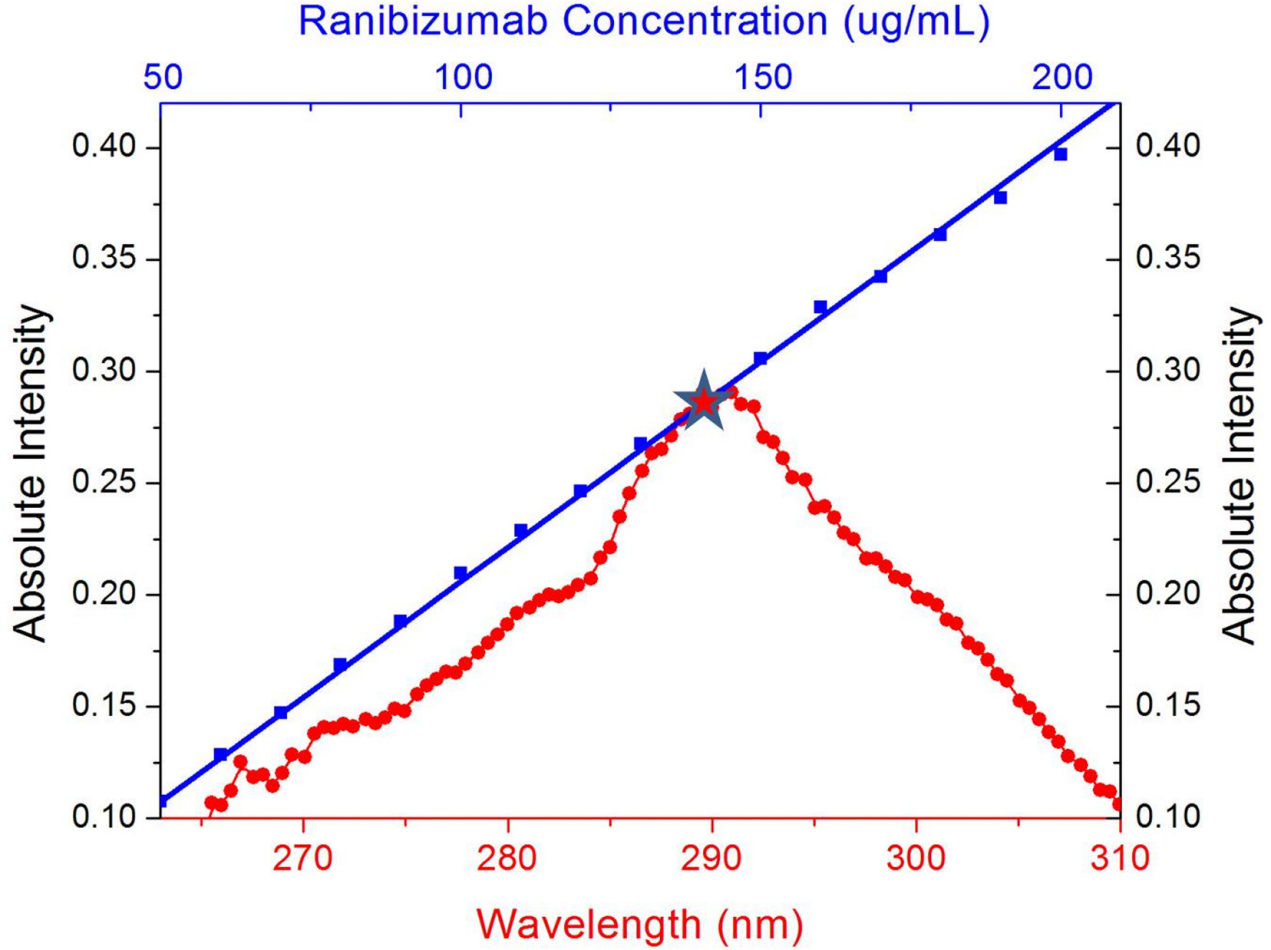
The UV-VIS spectrum of ranibizumab encapsulated MPs (red circles) and the standard curve for UV-VIS spectrophotometer intensity versus the ranibizumab concentration (blue squares). Standard curve is built at wavelength of 290 nm. The ranibizumab encapsulated MPs have a peak intensity of 0.291 at 290.96 nm, corresponding to a ranibizumab concentration of about 140 μg/mL. This value is equivalent to an encapsulation rate of 70%.

Fig 5 plots the standard curve of ranibizumab at concentration levels ranging from 50 μg/mL to 200 μg/mL overlapped with the absorption spectrum of the MPs produced. According to the figure, the ranibizumab-loaded MPs have a peak intensity of 0.291 at 290.96 nm, corresponding to a ranibizumab concentration of about 140 μg/mL, equivalent to an encapsulation rate of 70%.

**Fig 6:**
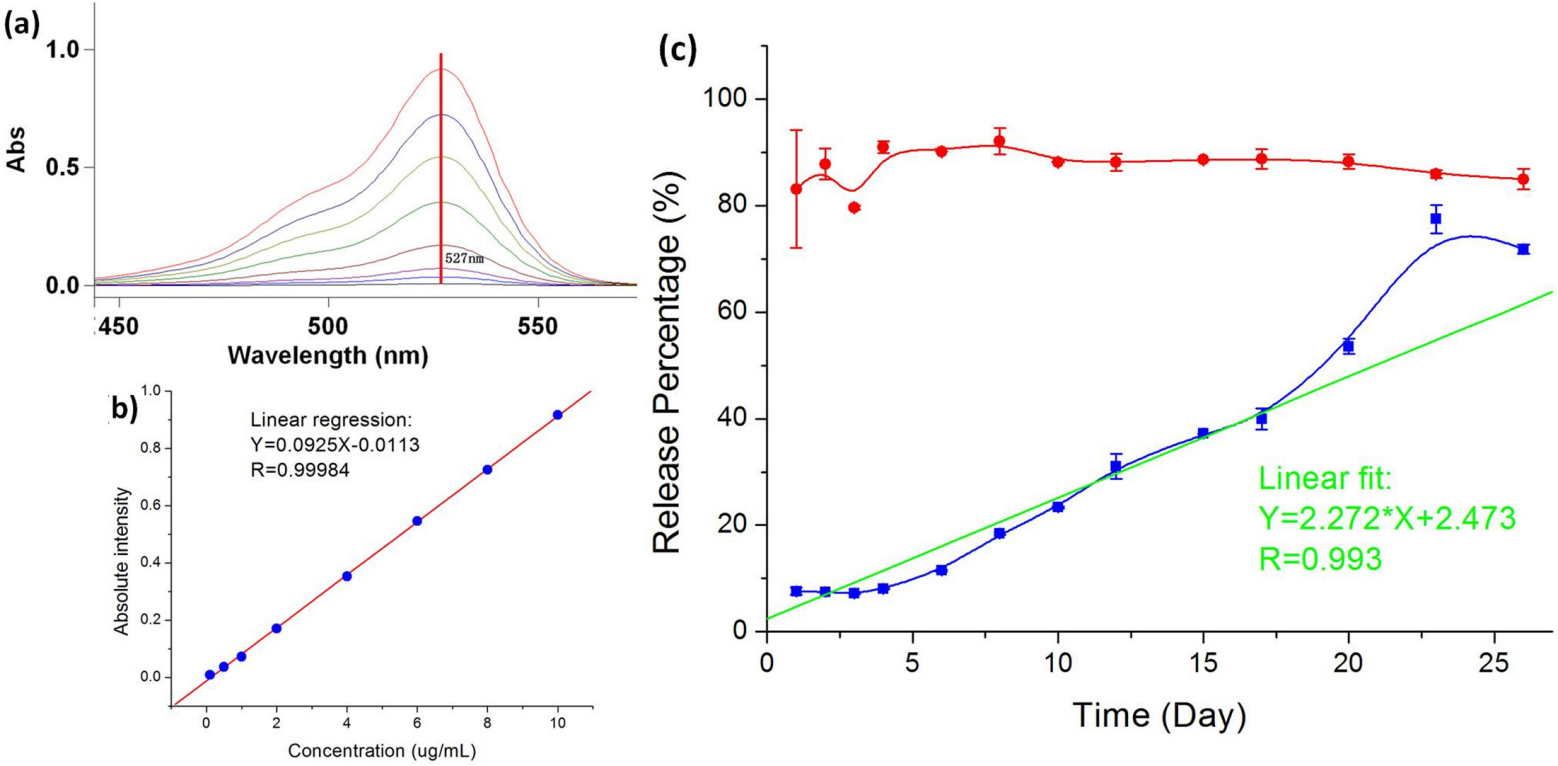
*In vitro* release profile of Rhodamine-6G and Rhodamine-6G encapsulated MPs in saline. (a) The changes of UV-VIS spectrometer intensity versus Rhodamine-6G concentration. Absorbance at 527 nm is used to build the standard curve. (b) Linear relationship between the UV-VIS spectrometer intensity versus Rhodamine-6G concentration. (c) *In vitro* release profile of Rhodamine-6G and Rhodamine-6G encapsulated MPs in saline. Red circles and the corresponding curve represent the concentration percentage of pure Rhodamine-6G in saline on various days as the control group. Blue rectangles and the corresponding curve represent the concentration percentage of Rhodamine-6G encapsulated MPs in saline on various days. Green line is the linear fit of release profile of Rhodamine-6G encapsulated MPs in saline. The experimental data are presented as mean ± SD from three sample tests at the same conditions.

The release profile of the drug-loaded MPs is tested using Rhodamine-6G as a model drug. Fig 6 plots the percentage of Rhodamine-6G released from the MPs over 20 days in comparison with the control solution of Rhodamine-6G dye at the same concentration. According to the figure, the core-shell structure of the MPs produced by the CES process can effectively control the drug release at a stable rate while direct application of the model drug without MP encapsulation does not yield a sustained drug release profile.

**Fig 7:**
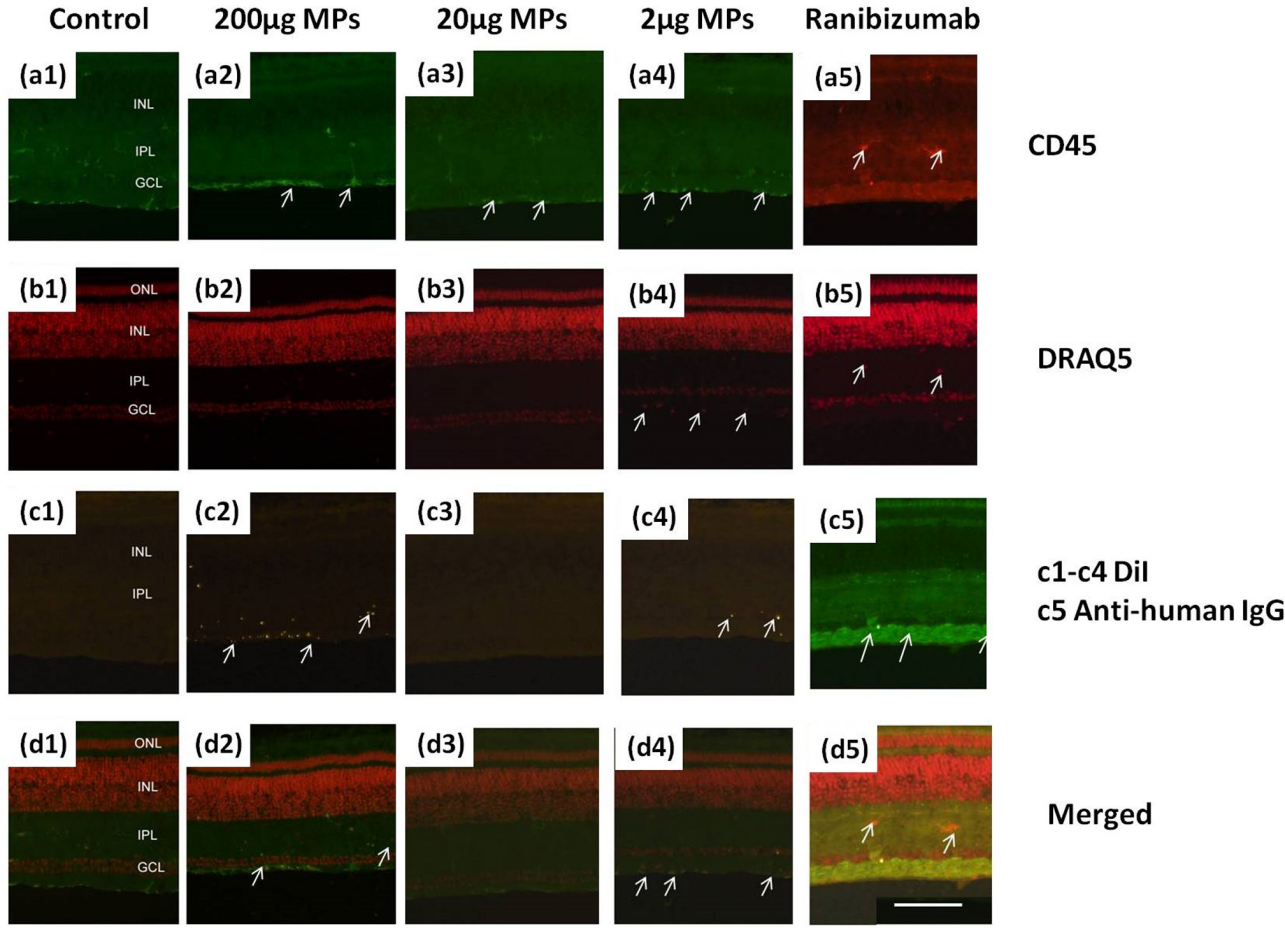
Microglia reaction of MPs and ranibizumab injection. **Retinas are harvested from eyes that are one day after injection.** Sections from left to right: (1) 20 μL saline+BSA as control; (2) 200μg/20μL MPs; (3) 20μg/20μL MPs; (4) 2μg/20μL MPs; (5) 200μg/20μL ranibizumab. Sections from top to bottom are labeled or stained with: (a) Mouse anti-CD45; (b) DRAQ5; (c1-c4) DiI; (c5) Goat anti-human IgG1; (d) Merged images. The calibration bar (100 μm) in panel (d5) applies to all images.

Microglia reaction after intravitreous injection of the drug-loaded MPs is examined in an *in vivo* chick model. CD45 is used to track the microglia’s activity in retina and DRAQ5 is used to label the nuclear DNA. Fig 7 shows the fluorescence microscopic images of the control and the sample retina tissue after staining. In comparison to the control retina, the microglia under 200 μg MPs injection is over-reactive. The pixel intensity and the density of CD45 immunofluorescence significantly increase after MP injection. The accumulation of CD45 immunofluorescence at the inner limiting membrane (ILM) is noticeable (arrows in Fig 7-a2). Moreover, DiI encapsulated MPs are observed in various retina layers, including the ganglion cell layer (GCL), inner plexiform layer (IPL), and even in inner nuclear layer (INL) (arrows in Fig 7-c2). MPs are also accumulated in the ILM layer with higher microglia density. However, the microglia is much less reactive after intravitreous injection of 20 μg and 2 μg MPs. Several MPs can be seen in the retina (arrows in Fig 7-c4), but not as noticeable as the 200 μg injection. Based on the pixel intensity and the density of CD45 immunofluorescence, there is no evidence that microglia is significantly reactive after intravitreous injection of 20 μg or 2 μg MPs. Meanwhile, ranibizumab is directly injected intravitrously and the retina tissue is collected and triple-labeled by mouse anti-human IgG1, CD45, and Draq5 (Fig 7-5). Compare to the control sample (Fig 7-c1), ranibizumab is accumulated in the GCL after one day from injection (arrows in Fig 7-c5). According to Fig 7(a5) and 7(b5), the microglia activity is slightly reactive but not significant after the injection of ranibizumab, further proving the clinical safety of ranibizumab injection from the immunocytochemical aspect.

**Fig 8:**
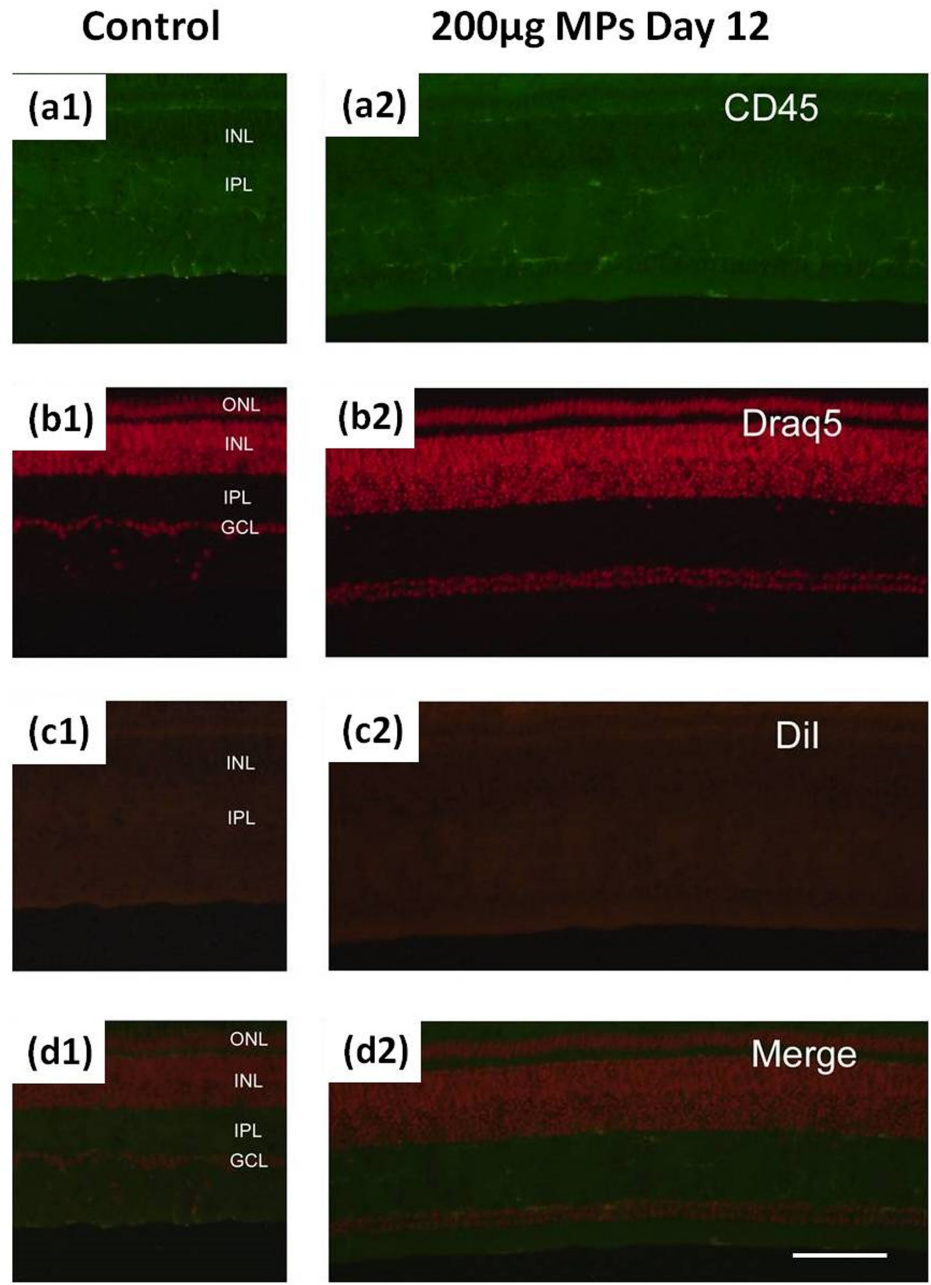
Microglia reaction of 200μg/20μL MPs injection. **Retinas are harvested from eyes that are twelve days after injection.** Sections from left to right: (1) 20 μL saline+BSA as control; (2) 200μg/20μL MPs. Sections from top to bottom are labeled or stained with: (a) Mouse anti-CD45; (b) DRAQ5; (c) DiI; (d) Merged images. The calibration bar (100 μm) in panel (d2) applies to all images.

Fig 8 shows the fluorescence microscopic images of the retina tissue harvested on the 12th day after intravitreous injection of MPs. No significant microglia reactions are observed for all 200 μg, 20 μg, and 2 μg dose groups in comparison with that of the control retina tissue. Unlike the short term reaction after the injection of 200 μg MPs, the MP deposition in retina is almost negligible and the microglia activity resumes the normal condition.

**Fig 9:**
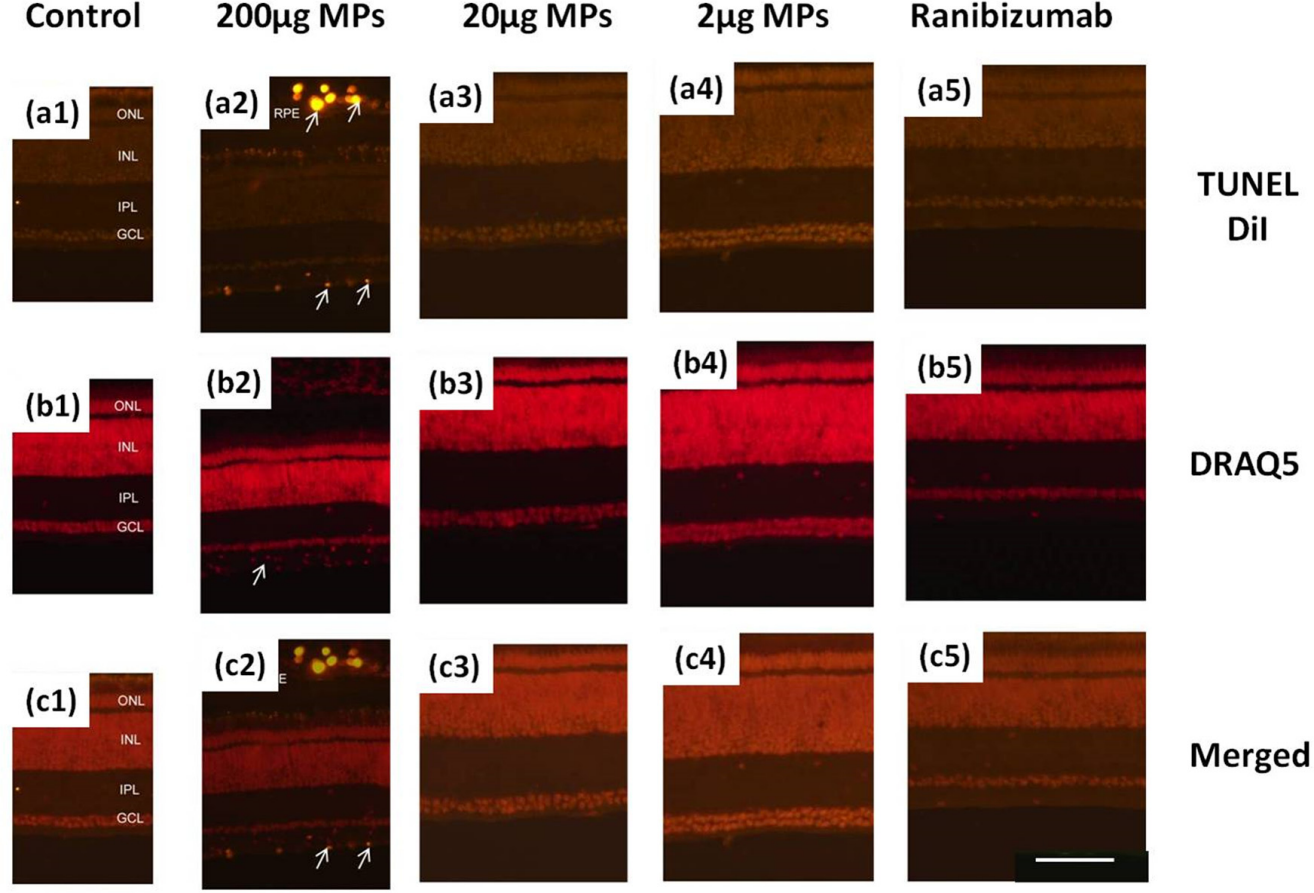
TUNEL cell death experiment of MPs and ranibizumab injection. **Retinas are harvested from eyes that are one day after injection.** Sections from left to right: (1) 20 μL saline+BSA as control; (2) 200μg/20μL MPs; (3) 20μg/20μL MPs; (4) 2μg/20μL MPs; (5) 200μg/20μL ranibizumab. Sections from top to bottom are labeled or stained with: (a) Dual channel for TUNEL and DiI; (b) DRAQ5; (c) Merged images. The calibration bar (100 μm) in panel (c5) applies to all images.

Considering that the microglia reaction reflects only the normal immune reaction to external stimulations, it does not necessarily indicate the cell death. To further evaluate the cell death after injection, the *In Situ* Cell Death Kit (TUNEL) is used. The retinal tissue after intravitreous injection of 200 μg ranibizumab is first labeled by TUNEL kit to verify that the current clinical procedure does not cause cell death. The slides are also labeled by Draq5 to observe the nuclei in the retina. As expected, there is no cell death with ranibizumab injection (Fig 9-5). In the result of short-term 200 μg MPs injection (Fig 9-2), we observe significant cell death in ILM (arrows in ILM in Fig 9-a2) along with nuclei accumulation in that layer (arrow in Fig 9-b2). We can conclude from the TUNEL result that intravitreous injection of 200 μg MPs causes cell death. Further, DiI encapsulated MPs are also observed in the blood vessel in outer retina (chorocapillaries and/or medium sized vessels), indicating that the MPs are transferred by immune system to protect the retina. With the evidence of microglia accumulation in ILM, we hypothesize that the microglia acts as phagocytic cell to transfer the MPs. Further study is need to confirm the actual mechanism. As the MPs are barely observed in the retina after intravitreous injection of 20 μg and 2 μg MPs, we can hardly observe the cell death in these retina samples. Moreover, the TUNEL assay run long-term after injecting 200 μg MPs does not find any evidence of cell death.

## Conclusion and Perspectives

Ranibizumab-loaded PLGA MPs are fabricated by an improved CES process. The core-shell structure is clearly observed by confocal fluorescence microscopy. Theoretically speaking, the process is able to achieve 100% encapsulation efficiency for water-soluble drugs and proteins. Practically, an encapsulation efficiency of around 70% is achieved in this study. The possible reasons for the reduced encapsulation efficiency are the uncollected stray droplets during the process and the loss of cargo materials during post-processing. Further enhancement of the encapsulation efficiency can be achieved by optimizing the process parameters. Our in vitro release test shows that the produced MPs facilitate long term sustained drug release in comparison with drugs without encapsulation. The inflammation and the retinal cell death after intravitreal injection of MPs are evaluated by the immunocytochemical technique using a chick model. No significant short-term or long-term microglia reactions are observed after intravitreous injection of 2 μg MPs, 20 μg MPs, or ranibizumab. Apoptosis of cell death is neither observed after these injections. However, the significant microglia reaction as well as the cell death (labeled by TUNEL) is observed short-term after injection of 200 μg MPs, but no long-term reaction is observed. Our study demonstrates the technical feasibility of using the CES process as a microencapsulation method to load water-soluble drugs for sustained release of anti-VEGF therapeutics in the treatment of AMD. In addition to wet AMD, the application of this technology may ultimately play a large role in all retinal vascular diseases as anti-VEGF drugs have been approved by FDA for uses in other ocular diseases such as diabetic macular edema, retinal edema, and central retinal vein occlusion, and for off-label uses in numerous other conditions where VEGF is the mediator for retinal edema and angiogenic complications.

Although CES is a promising process with a great potential, the technology is still at its early stage and requires further research and development. The works we have done in CES is based on individual lab experience, specific material combinations, and empirical process parameters. Standard protocols and systematic process control are needed for reliable and reproducible fabrication of multi-layered MPs. A more effective MP collection method and a more productive needle design are necessary for mass production of the multi-layered MPs. On the physiological side, further experiments are needed in order to evaluate the biologic responses of intravitreous MP delivery and its intrinsic mechanism. First, more time points are needed for the harvest of retinal tissue after injection in order to understand how MPs are migrated from vitreous to the blood vessels through the retina. Second, more glia cells, such as Muller glia, should be investigated to fully understand the immune reaction after intravitreous injection of MPs. Third, the neurons, especially the optic nerve, should be labeled to investigate if there is damage to the neurons after MP injection. Finally, it is still unclear about the upper limit of the MP dose that may introduce the long term adverse effects on retina tissue, although we may conclude from our experiment that intravitreous injection of 20 μg MPs does not introduce significant microglia reaction while 200 μg MPs does. Therefore, further animal validation tests and toxicology studies are necessary in order to determine the specific dosage threshold for MP injection that does not induce inflammation and retinal cell death.
